# Development of a TaqMan probe-based multiplex real-time PCR for the simultaneous detection of four clinically important filamentous fungi

**DOI:** 10.1128/spectrum.00634-24

**Published:** 2024-07-30

**Authors:** Yutong Wei, Yangxuan Lin, Jingya Zhao, Dingchen Li, Zhankui Yang, Fangyan Chen, Li Han

**Affiliations:** 1 School of Public Health, Anhui Medical University, Anhui, China; 2 Department for Disinfection and Infection Control, Chinese PLA Center for Disease Control and Prevention, Beijing, China; 3 Zhengzhou University, Zhengzhou, China; University of Debrecen, Debrecen, Hungary

**Keywords:** fungal infection, multiplex real-time qPCR, *Aspergillus fumigatus*, Mucorales, *Histoplasma capsulatum*, *Fusarium* spp

## Abstract

Filamentous fungi present significant health hazards to immunocompromised individuals globally; however, the prompt and precise identification of them during infection remains challenging. In this study, a TaqMan probe-based multiplex real-time PCR (M-qPCR) assay was developed to detect simultaneously the target genes of four important pathogenic filamentous fungi: *ANXC4* gene of *Aspergillus fumigatus, EF1-α* gene of *Fusarium* spp*.*, mitochondrial *rnl* gene of Mucorales, and *hcp100* gene of *Histoplasma capsulatum*. In this M-qPCR assay, the limit of detection (LoD) to all four kinds of fungi was 100 copies and the correlation coefficients (R^2^) were above 0.99. The specificity of this assay is 100%, and the minimum detection limit is 100 copies/reaction. In conclusion, an M-qPCR detection assay was well established with high specificity and sensitivity for rapid and simultaneous detection on four important filamentous fungi in the clinic.

**IMPORTANCE:**

World Health Organization developed the first fungal priority pathogens list (WHO FPPL) in 2022. *Aspergillus fumigatus*, Mucorales, *Fusarium* spp., and *Histoplasma* spp. are the four types of pathogenic fungi with filamentous morphology in the critical priority group and high priority group of WHO FPPL. These four filamentous fungal infections have become more common and severe in immunocompromised patients with the increase in susceptible populations in recent decades, which resulted in a substantial burden on the public health system. However, prompt and precise identification of them during infection remains challenging. Our study established successfully a TaqMan probe-based multiplex real-time qPCR assay for four clinically important filamentous fungi, *A. fumigatus*, *Fusarium* spp., Mucorales, and *Histoplasma capsulatum,* with high sensitivity and specificity, which shows promising potential for prompt and precise diagnosis against fungal infection.

## INTRODUCTION

Fungi are ubiquitous eukaryotic organisms in the environment (1). To date, several hundred of fungal species have been known to be capable of causing various human infectious diseases (2, 3). Globally, each year, approximately 3 million people develop chronic severe fungal infections and nearly 1.9 million patients develop acute invasive fungal disease (IFD) (4). With the mortality rate ranging from 30% to 90%, invasive fungal infections have become more common and severe in immunocompromised patients with the increase in susceptible populations in recent decades (5), which resulted in a substantial burden on the public health system (3, 6).

Significantly, in response to the rising threat of fungal infections, combined with existing and emerging resistance and treatability issues, the World Health Organization (WHO) developed the first fungal priority pathogens list (WHO FPPL) in 2022 (7). *Aspergillus fumigatus*, Mucorales, *Fusarium* spp., and *Histoplasma* spp. are the four types of pathogenic fungi with filamentous morphology in the critical priority group and high priority group of WHO FPPL. *A. fumigatus* is the primary pathogen for invasive aspergillosis (IA), chronic aspergillosis, and allergic bronchopulmonary aspergillosis, etc., through the inhalation of its ubiquitous conidia by host. Recently, it has also been observed in patients with severe influenza or COVID-19 pneumonia (8). This study revealed a significant occurrence of IA exceeding 10% among intensive care unit (ICU) patients with influenza pneumonia, and this condition was linked to elevated mortality rates in critically ill individuals infected with COVID-19, ranging from 16% to 25% (9). Mucormycosis, a life-threatening infection primarily caused by the genus *Rhizopus* of the order Mucorales, is a rare and challenging-to-diagnose disease associated with considerable morbidity and mortality (8, 10). Furthermore, the mortality rate of mucormycosis varies between 40% and 80%, depending on the underlying conditions and sites of infection (10). Fusariosis ranks as the third most prevalent mold infection in many countries (10, 11), trailing behind aspergillosis and mucormycosis. In immunocompromised patients, infections caused by *Fusarium* spp. exhibit a substantial mortality rate, ranging from 50% to 70% (11, 12). Histoplasmosis, attributed to *Histoplasma* spp., presents a wide range of clinical manifestations, spanning from subclinical or mild respiratory disease to progressive disseminated histoplasmosis (PDH). It stands as a prominent cause of fungal respiratory infection in regions where it is endemic (13, 14); in many highly endemic countries, accurate diagnosis of histoplasmosis remains challenging and limited (15).

The scarcity of prompt and accurate diagnostic techniques significantly contributes to the elevated morbidity and mortality associated with fungal infections. The sensitivity of microscopic examination for detecting fungal infections is limited, particularly in the early stages of infection or during the optimal treatment window (5, 16). Although culture methods are considered the gold standard for identifying fungal pathogens, they are time-consuming with low positivity rate, which significantly hinders the prompt and effective treatment for patients.

With the advancement of molecular biology and sequencing technology, quantitative real‐time reverse transcription polymerase chain reaction (RT-qPCR) assay has been widely used for the detection of fungi. It has been applied in diagnosing histoplasmosis from blood and bronchoalveolar lavage (17), as well as accurately identifying clinically important fungal genera and species, such as *Aspergillus* spp., *Fusarium* spp., *Scedosporium,* and the *Mucormycetes* from paraffin-embedded tissues (18). Multiplex qPCR (M-qPCR) assay had been developed for the simultaneous and precise detection of multiple targets in a single reaction, thereby improving the speed and efficiency of diagnosis. For fungal detection, several M-qPCR assays were developed for the simultaneous detection of three types of fungi, including *Sporothrix brasiliensis, Sporothrix globose, and Sporothrix schenckii* (19), and the identification of *Pneumocystis jirovecii*, *H. capsulatum*, and *Cryptococcus neoformans*/*Cryptococcus gattii* in samples obtained from AIDS patients suffering from opportunistic pneumonia (20).

Up to date, no M-qPCR method was available for the simultaneous detection of four clinically important filamentous fungi, *A. fumigatus*, Mucorales, *Fusarium* spp., and *Histoplasma* spp. listed in the critical priority group and high priority group of WHO FPPL. In this study, a TaqMan probe-based M-qPCR assay was developed to simultaneously and specifically detect these four types of fungi for prompt clinical detection.

## MATERIALS AND METHODS

### Strains and growth conditions

The strains utilized in this research were obtained from the BeNa Culture Collection, China Center of Industrial Culture Collection, and the laboratory of the Department of Disinfection and Infection Control, Chinese PLA Center for Disease Control and Prevention (as indicated in Table 1). The fungi were cultivated on potato dextrose agar at a temperature of 28°C or 37°C and allowed to incubate for 2–5 days. Bacteria were cultured in Luria-Bertani (LB) liquid culture medium at 37°C on a shaker at 60 r/min overnight.

**TABLE 1:** Strains used in this study

Genus	Latin name	Source/strain
*Aspergillus*	*Aspergillus fumigatus*	B5233
*Fusarium*	*Fusarium solani*	BNCC337554
	*Fusarium oxysporum*	BNCC120618
	*Fusarium fujikuroi*	BNCC186248
Mucorales	*Rhizopus microsporus*	BNCC145129
	*Rhizopus oryzae*	BNCC336269
	*Mucor racemosus*	BNCC336225
	*Mucor circinelloides*	BNCC147484
*Histoplasma*	*Histoplasma capsulatum*	Isolated strain
Non-targeted	*Penicillium oxalicum*	Isolated strain
	*Penicillium chrysogenum*	Isolated strain
	*Aspergillus flavus*	Isolated strain
	*Aspergillus terreus*	Isolated strain
	*Aspergillus niger*	Isolated strain
	*Candida albicans*	Isolated strain
	*Candida auris*	Isolated strain
	*Candida parapsilosis*	Isolated strain
	*Candida glabrata*	Isolated strain
	*Cryptococcus neoformans*	Isolated strain
	*Cryptococcus gattii*	Isolated strain
	*Staphylococcus aureus*	Isolated strain
	*Escherichia coli*	Isolated strain
	Type II human alveolar epithelial cell	A549

### Primer and probe design

The species-specific qPCR primers and probe sets used in this study for Mucorales were developed by Rui Xu (21), the detection target is the mitochondrial gene *rnl*, and the primers and probes for *A. fumigatus*, *Fusarium* spp., and *H. capsulatum* were designed and developed by software AlleleID 6.0 (Primer BioSoft, USA) in this study. The detection target selection for *A. fumigatus*, *Fusarium* spp., and *H. capsulatum* is annexin (*ANXC4*) gene, translation elongation factor 1-alpha (*EF1-α*) gene, and 100 kDa protein (*Hcp100*) gene, respectively.

Details of the target gene name, primers, and probes are listed in Table 2. The pairwise sequence alignment tool of the Basic Local Alignment Search Tool (BLAST) from the National Center for Biotechnology Information (NCBI) was used to analyze the specificity of primers and probes, confirming the absence of cross-reactivity with human and non-target fungi. All primers and probes were synthesized by Sangon Biotech (Shanghai, China).

**TABLE 2:** Primer and TaqMan probe sequences used in qPCR assay

Organism	Gene target	Oligonucleotide (5’−3’)	Final reaction concentration (nM)
*A. fumigatus*	Annexin C4 (*ANXC4*)	Forward: CAGTGACGTATGAGAGTC	200
		Reverse: GGACATAACTGGACCATC	200
		Probe: FAM-CTACTCAGATACGGACGACGAG-BHQ1	100
Mucorales	*rnl*	Forward: AACCGACACTGGTCTGCTG	200
		Reverse: TCTTCTATTCTGTGCCACGAC	200
		Probe: VIC-TCCCGAAGTTACGGAGTCATTTTGC- BHQ1	100
*H. capsulatum*	100 kDa protein (*Hcp100*)	Forward: TTCCGTGCAGAAAATTCG	200
		Reverse: GATCCAATGTCCGTTCAC	200
		Probe: Texas Red-TCCCGAAGTTACGGAGTCATTTTGC-BHQ2	100
*Fusarium*	Translation elongation factor 1 alpha (*EF1-α*)	Forward: GGTAAGGAGGACAAGACTCACC	200
		Reverse: TTGTCGATACCACCGCACTG	200
		Probe: CY5-CGTCGTCGTCATCGGCCACGTCG-BHQ2	100

### DNA extraction

Genomic DNA of all fungal/bacterial cultures was extracted using the Biospin Fungal/bacterial genomic DNA Extraction kit (BioerTechnology, Hangzhou, China), and simulated clinical samples were processed using the DNeasy Blood and Tissue Kit (Qiagen, Madrid, Spain), according to the manufacturer’s instructions. Total elution volume of DNA was 50 µL. Quantification of DNA was performed using a DeNovix spectrophotometer (DeNovix Inc., USA). Samples were then stored at −20°C until analysis.

### Construction and extraction of recombinant plasmids

To obtain engineered bacteria containing the target gene, the *ANXC4* gene of *A. fumigatus*, *rnl* gene of Mucorales, *EF1-α* gene of *Fusarium* spp., and *Hcp100* gene of *H. capsulatum* were constructed into pUC57 vector by Sangon Biotech. A plasmid mini-extract kit (OMEGA company) was employed to extract the plasmid DNA containing the target gene. Quantification of DNA was performed using a DeNovix spectrophotometer (DeNovix Inc., USA). The copy number of recombination plasmids was calculated by using the following formula (22): plasmid copies/μL = (6.02 × 10^23^) × (ng/μL × 10^−9^)/[ plasmid length (bp) × 660].

Each plasmid was diluted from 1 × 10^10^ copies/μL to 1 × 10^1^ copies/μL with a 10-fold gradient dilution to establish a simplex standard curve for each type of fungus. For the construction of multiplex standard curves, the plasmids of four fungi were mixed with equal mass and then diluted 10-fold gradient to 1 × 10^1^ copies/μL. All plasmid DNA was then stored at −20°C until analysis.

### Real-time qPCR assay

The real-time qPCR reaction was conducted in a 20 µL volume and performed in the Bio-Rad CFX Opus 96 System (Bio-Rad, CA, USA). For simplex qPCR (S-qPCR), the reaction mixture consisted of 10 µL 2× FsatFire qPCR Pre-Mix (Beijing Tiangen, China), 0.8 µL of each primer(10 µM), 0.4 µL of probe(10 µM), 7 µL nuclease-free water, and 1 µL DNA template. For multiplex qPCR (M-qPCR), the reaction mixture consisted of 10 µL 2× FsatFire qPCR Pre-Mix combined with all primers, probes, templates, and nuclease-free water. The concentrations of each primer and probe of *A. fumigatus*, Mucorales, *Fusarium* spp., and *H. capsulatum* were optimized for better outputs.

The cycling conditions encompassed an initial preincubation step at 95°C for 1 min, followed by an amplification program consisting of 40 cycles: denaturation at 95°C for 5 s and annealing/extension at 60°C for 15 s. The amplifying cycles of M-qPCR were carried out as the same as S-qPCR. The threshold for determining positivity was established as a cycle threshold (CT) value of <38. The quantification cycle values were assessed for each reaction using CFX Maestro software. In each experimental trial, both negative and positive controls were included.

### Specificity, sensitivity, and reproducibility analysis of qPCR assay

Specificity and potential cross-reactivity were evaluated by using four target plasmids (*A. fumigatus*, Mucorales, *H. capsulatum*, and *Fusarium* spp.) and genomic DNA extracted from 13 non-target strains (Table 1) and type II human alveolar epithelial cell A549. Nuclease-free water was used as blank control. Each experiment was repeated three times.

To analyze the sensitivity of M-qPCR, 10-fold dilutions of mixtures from the four plasmids were prepared as above, with the final concentration ranging from 1 × 10^10^ copies/μL to 1 × 10^1^ copies/μL. Each experiment was repeated three times.

The repeatability analysis of M-qPCR was assessed by intra-assay and inter-assay, and 10-fold dilutions of mixtures from the four plasmids were used as templates for amplification.

### Validation of M-qPCR assay

The substrates used for the simulation experiments were artificial sputum medium and simulated serum (Biochemazone, Canada). To simulate clinical samples, a 10-fold dilution series of 10^6^ to 10^1^ conidia of *A. fumigatus*, three *Fusarium* species (*F. solani*, *F. oxysporum*, and *F. fujikuroi*) and sporangiospores of Mucorales (*R. microsporus, R. oryzae, M. racemosus,* and *M. circinelloides*) were added to 200 µL simulated serum and sputum, respectively. The genomic DNA was extracted as described above. Due to the fact that cultivation of *H. capsulatum* requires a biological safety protection third-level laboratory, we added 2 mg to 20 ng mycelium powder of *H. capsulatum* to simulated serum and sputum as simulated clinical samples for M-qPCR assay, and the genomic DNA was extracted as mentioned above. All the genomic DNA was stored at −20°C until analysis.

### Statistical analysis

Statistical analysis was conducted using GraphPad Prism8 software (GraphPad Software, La Jolla, CA). The data in Table 4 were represented as the mean ± standard deviation of the mean from at least three independent experiments, whereas the data in Fig. 2 and 4 were represented as the mean. A linear regression analysis was used to assess the differences between the S-qPCR and M-qPCR standard curves. Variability was measured by the standard error of the mean.

## RESULTS

### Specificity of the S-qPCR assays

**Fig 1:**
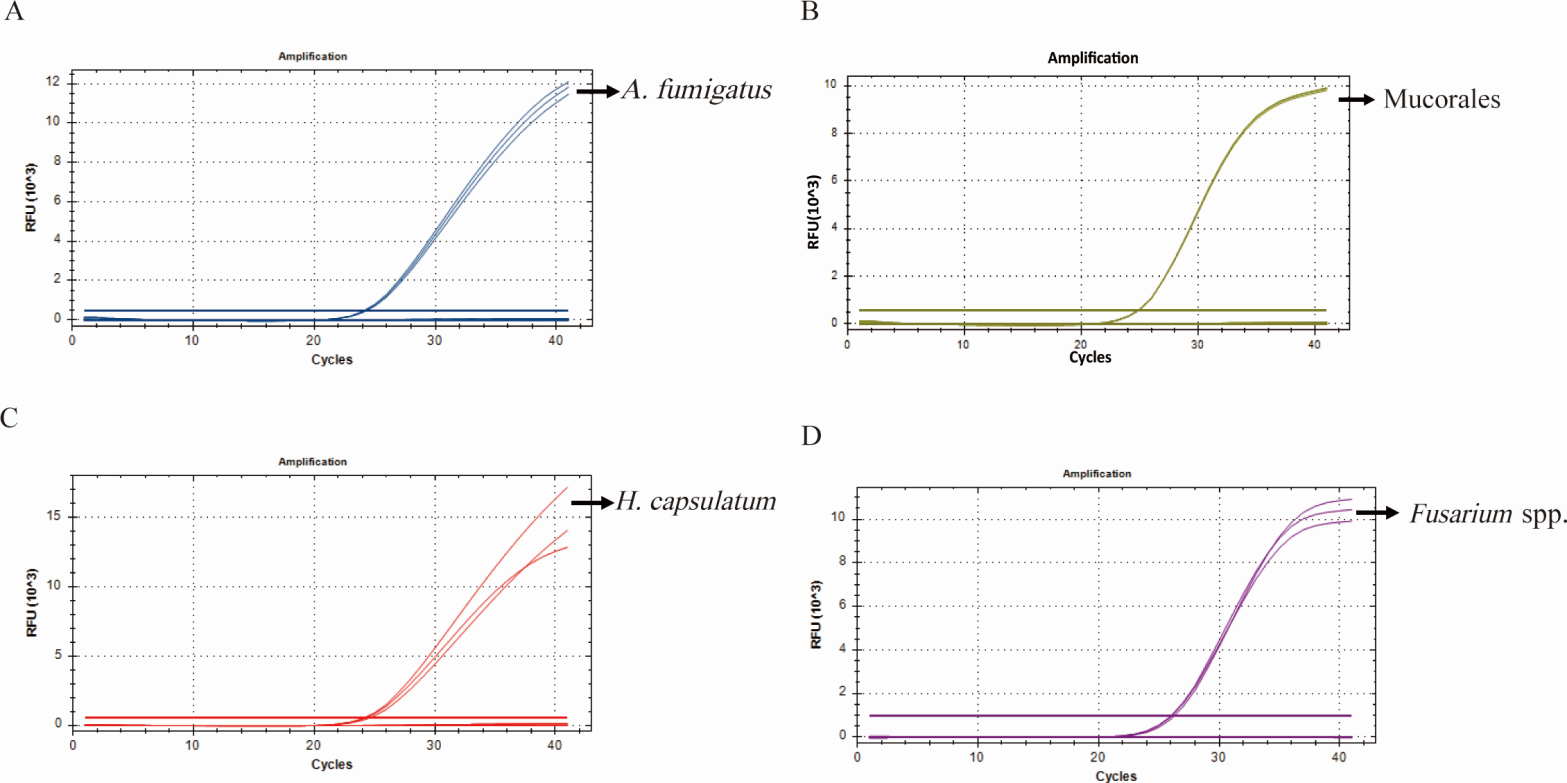
Specificity of primers and probes of four fungi determined by S-qPCR assay. Plasmid DNA from *A. fumigatus* (A), Mucorales (B), *H. capsulatum* (C), and *Fusarium* spp. (D) and genomic DNA from 13 non-target fungi and type II human alveolar epithelial cell A549 were used as the template for S-qPCR reaction mixtures.

The primer and probe for each target of four fungi, *A. fumigatus*, M ucorales, *H. capsulatum*, and *Fusarium* spp. were established and evaluated by S-qPCR assay, respectively. The specificity of S-qPCR was confirmed with plasmid DNA of these four fungi, and genomic DNA was extracted from 13 non-target strains (Table 1) and A549. As shown in Fig. 1A, *A*. *fumigatus* sample exhibited the amplification curve in S-qPCR reaction, which included the primers and probes (Table 2) targeting *ANXC4* gene of *A. fumigatus*, whereas the 16 non-*A*. *fumigatus* samples and the human blood sample did not yield amplification (Table S3). As shown in Fig. 1B, only the Mucorales sample exhibited the amplification curve in S-qPCR reaction, which included the primers and probes (Table 2) targeting *rnl* gene of Mucorales, whereas the 16 non-Mucorales samples and the human blood sample did not yield amplification (Table S3). As shown in Fig. 1C, only *H. capsulatum* sample exhibited the amplification curve in S-qPCR reaction, which included the primers and probes (Table 2) targeting *Hcp100* gene of *H. capsulatum*, whereas the 16 non-*H*. *capsulatum* samples and the A549 did not yield amplification (Table S3). As shown in Fig. 1D, only the *Fusarium* spp. sample exhibited the amplification curve in S-qPCR reaction, which included the primers and probes (Table 2) targeting the *EF1-α* gene of *Fusarium* spp., whereas the 16 non-*Fusarium* spp. samples and the A549 did not yield amplification (Table S3). All the amplification curves were smooth and S-shaped.

### Standard curves of S-qPCR assay

**Fig 2:**
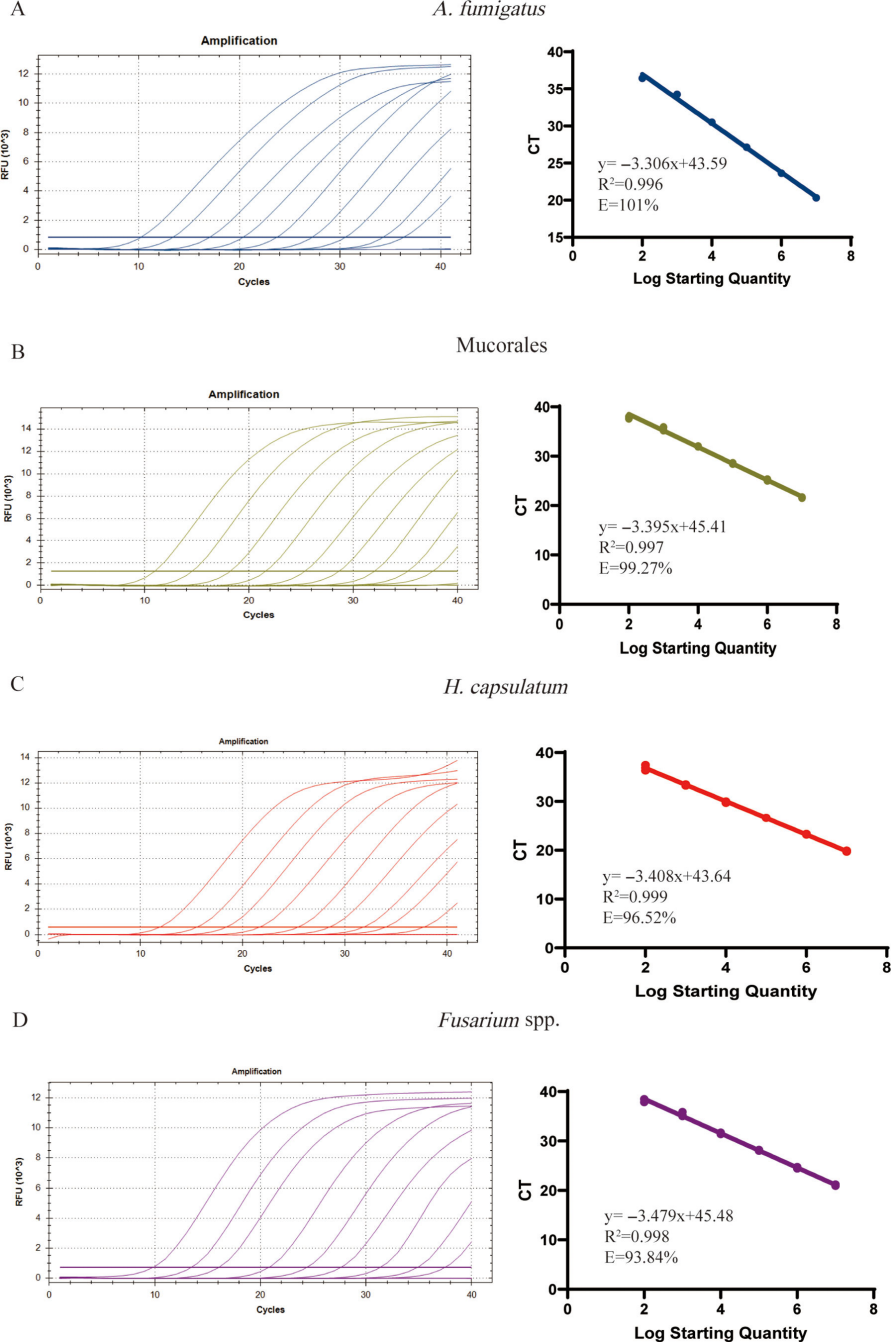
The standard curve of *A. fumigatus*, Mucorales, *H. capsulatum,* and *Fusarium* spp. by S-qPCR assay. The standard curve was established using the obtained CT values as the vertical coordinates and the logarithm of the concentration of the plasmid as the horizontal coordinate (10^2^–10^7^ copies/μL). *A. fumigatus* (A), Mucorales (B), *H. capsulatum* (C), and *Fusarium* spp. (D). All data points are from an average of three technical replicates.

The efficiency of S-qPCR for each target was investigated by constructing a standard curve based on the serial dilution of plasmid DNA. The standard curves showed linearity for *A. fumigatus* (Fig. 2A), with high efficiencies of 101%, 99.27% for Mucorales (Fig. 2B), 96.52% for *H. capsulatum* (Fig. 2C), and 93.84% for *Fusarium* spp. (Fig. 2D). The amplification curves of the four fungi had a good linear relationship with the CT values, with all R^2^ values above 0.99. All target species were detected at the minimum plasmid DNA concentration of 1 × 10^2^ copies/μL, corresponding with CT values of 36.5 ± 0.26 for *A. fumigatus*, 37.79 ± 0.26 for Mucorales, 37.18 ± 0.41 for *H. capsulatum*, and 37.51 ± 0.49 for *Fusarium* spp. (Table S1).

### Optimization of M-qPCR reaction conditions

In order to optimize the reaction condition of M-qPCR assay, the concentration of each primer and probe in the whole reaction was combined using an orthogonal design (Table S2). In total, 25 different combinations were tested, and the lowest plasmid DNA concentration detected was in group 13. Taken together, the concentrations of primers and probes for four fungi ultimately applied to M-qPCR reaction were all 100 nM and 200 nM, respectively.

### Specificity of M-qPCR assay

**Fig 3:**
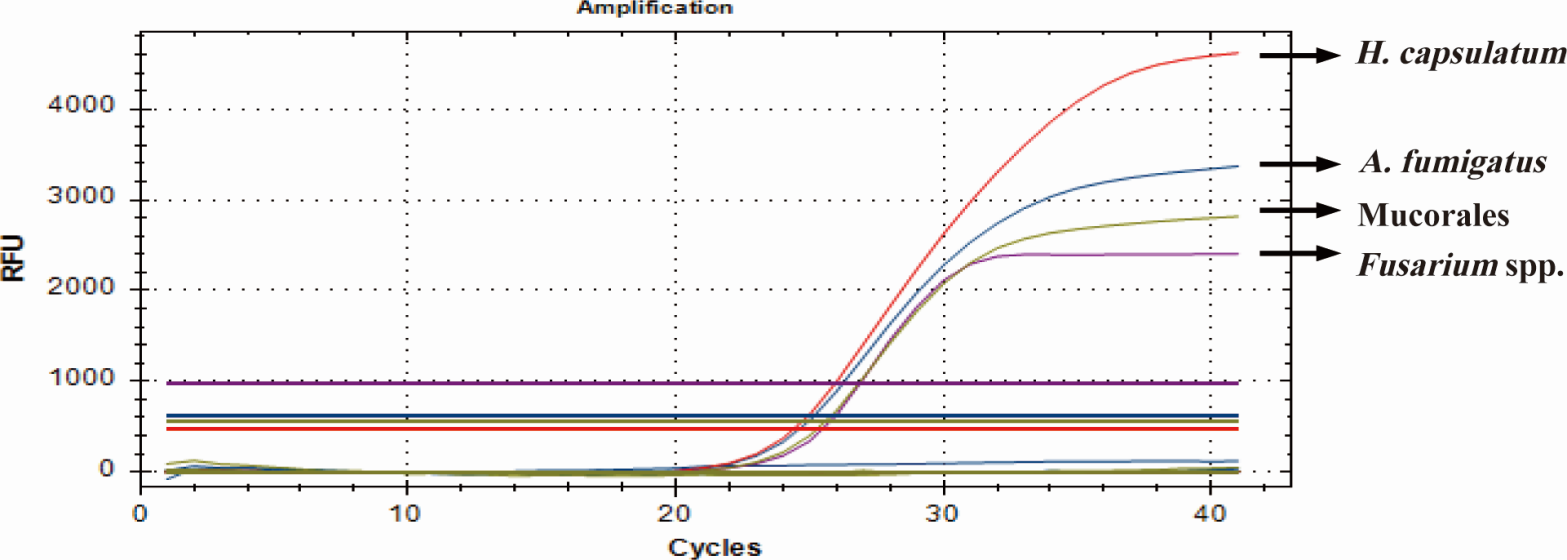
Specificity analysis of M-qPCR assay. Positive control from four plasmids targeted to *A. fumigatus* (blue), Mucorales (green), *H. capsulatum* (red), and *Fusarium* spp. (purple) and genomic DNA from 13 non-target strains and type II human alveolar epithelial cell A549 were used as templates for M-qPCR assay.

The specificity of M-qPCR was testified in the above optimal reaction system with DNA templates as the same as S-qPCR. As shown in Fig. 3, the targeted genes of four fungi were amplified simultaneously and separately with a slight variation in CT values in comparison to the amplifications in S-qPCR, whereas the 13 non-target strains and A549 did not yield any amplification (Table S4). The specificity and sensitivity of each fungus in M-qPCR were similar to those observed in S-qPCR reactions.

### Standard curves of M-qPCR assay

**Fig 4:**
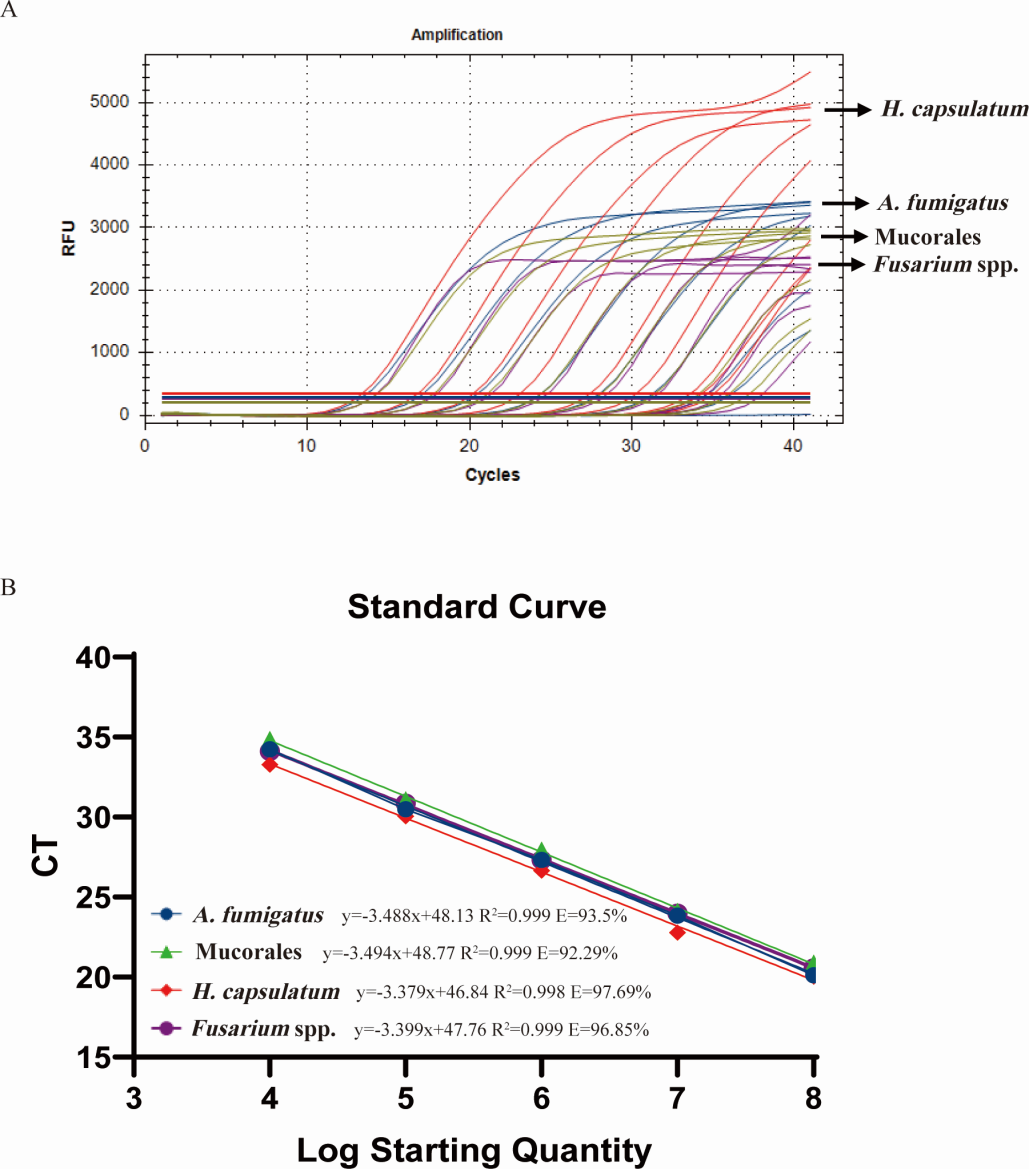
Sensitivity analysis and standard curves of M-qPCR. Concomitant amplification (**A**) and standard curves (10^4^–10^8^ copies/μL) (**B**) of four DNA targets by M-qPCR were illustrated and differentiated by *A. fumigatus* (blue), Mucorales (green), *H. capsulatum* (red), and *Fusarium* spp. (purple). All data points are from an average of three technical replicates.

To estimate the efficiency of M-qPCR, a standard curve was constructed based on the serial dilutions of mixed plasmid DNA to estimate the multiplex qPCR efficiency for each target. As shown in Fig. 4, all target species were detected at the minimum plasmid DNA concentration of 1 × 10^2^ copies/μL with similar CT values to those observed in S-qPCR. The standard curves of four fungi showed good linearity; the efficiency of *A. fumigatus* was 93.50 (R^2^ = 0.999), 92.29 for Mucorales (R^2^ = 0.999), 97.69 for *H. capsulatum* (R^2^ = 0.998), and 96.85 for *Fusarium* spp. (R^2^ = 0.999), respectively.

### Reproducibility of M-qPCR assay

The reproducibility of the M-qPCR assay was evaluated by intra-assay and inter-assay with the 10-fold serial dilutions of the mixed plasmid DNA (1 × 10^10^ copies/µL ~ 1 × 10^1^ copies/µL). For intra-assay, the plasmids with different dilutions were amplified three times simultaneously. For inter-assay, they were performed at three separate times with standards from different batches. As shown in Table 3, the standard deviation (SD) and coefficients of variation (CV) of CT values were from 0.02 to 0.53 and from 0.036% to 1.67% in intra-group, whereas they were from 0.08 to 1.62 and from 0.35% to 8.21% in inter-group, respectively. This indicated that the newly developed M-qPCR assay had good intra- and inter-reproducibility at the concentration from 1 × 10^2^ copies/µL to 1 × 10^10^ copies/µL.

**TABLE 3:** Analysis of the reproducibility of M-qPCR

Species	Copies/μL	Intra-assay		Inter-assay	
		CT (mean ± SD)	CV%	CT (mean ± SD)	CV%
*A. fumigatus*	1 × 10^1^	ND^*b*^	ND	ND	ND
	1 × 10^2^	36.66^*a*^	ND	36.95 ± 0.29	0.78
	1 × 10^3^	35.63 ± 0.53	1.49	36.04 ± 0.59	1.63
	1 × 10^4^	34.24 ± 0.49	1.43	34.03 ± 0.49	1.5
	1 × 10^5^	30.49 ± 0.09	0.3	30.57 ± 0.20	0.65
	1 × 10^6^	27.33 ± 0.29	1.06	27.29 ± 0.25	0.92
	1 × 10^7^	23.85 ± 0.08	0.34	23.97 ± 0.15	0.63
	1 × 10^8^	20.1 ± 0.05	0.25	19.63 ± 1.4	7.13
	1 × 10^9^	16.72 ± 0.28	1.67	16.73 ± 0.2	1.2
	1 × 10^10^	13.19 ± 0.05	0.38	13.24 ± 0.09	0.68
Mucorales	1 × 10^1^	ND	ND	ND	ND
	1 × 10^2^	37.01 ± 0.23	0.62	36.06 ± 0.88	2.44
	1 × 10^3^	35.4 ± 0.46	1.3	34.68 ± 0.0.82	2.36
	1 × 10^4^	34.2 ± 0.29	0.85	33.39 ± 0.68	2.04
	1 × 10^5^	31.01 ± 0.17	0.55	30.31 ± 0.56	1.85
	1 × 10^6^	27.7 ± 0.28	1.01	26.98 ± 0.62	2.3
	1 × 10^7^	24.12 ± 0.03	0.12	23.51 ± 0.5	2.13
	1 × 10^8^	20.91 ± 0.04	0.19	19.73 ± 1.62	8.21
	1 × 10^9^	17.57 ± 0.24	1.37	16.92 ± 0.54	3.19
	1 × 10^10^	13 ± 0.04	0.31	13.18 ± 0.2	1.52
*H. capsulatum*	1 × 10^1^	ND	ND	ND	ND
	1 × 10^2^	35.67 ± 0.13	0.036	35.38 ± 0.55	1.55
	1 × 10^3^	34.09 ± 0.07	0.21	33.66 ± 0.33	0.98
	1 × 10^4^	33.08 ± 0.41	1.24	33.38 ± 0.49	1.47
	1 × 10^5^	30.14 ± 0.19	0.63	30.11 ± 0.16	0.53
	1 × 10^6^	27.06 ± 0.21	0.78	26.86 ± 0.23	0.86
	1 × 10^7^	22.97 ± 0.06	0.26	22.86 ± 0.08	0.35
	1 × 10^8^	20.07 ± 0.06	0.3	19.98 ± 0.17	0.85
	1 × 10^9^	16.74 ± 0.27	1.61	16.57 ± 0.21	1.27
	1 × 10^10^	13.36 ± 0.04	0.3	13.24 ± 0.11	0.83
*Fusarium* spp.	1 × 10^1^	ND	ND	ND	ND
	1 × 10^2^	36.98 ± 0.36	0.97	37.14 ± 0.69	1.86
	1 × 10^3^	35 ± 0.41	1.17	35.33 ± 0.52	1.47
	1 × 10^4^	34.34 ± 0.13	0.38	34.25 ± 0.22	0.64
	1 × 10^5^	31.12 ± 0.17	0.55	31.04 ± 0.14	0.45
	1 × 10^6^	27.85 ± 0.2	0.72	27.68 ± 0.28	1.01
	1 × 10^7^	24.19 ± 0.05	0.21	24.16 ± 0.13	0.54
	1 × 10^8^	20.99 ± 0.04	0.19	20.79 ± 0.18	0.87
	1 × 10^9^	17.56 ± 0.25	1.42	17.4 ± 0.21	1.21
	1 × 10^10^	13.83 ± 0.02	0.14	13.77 ± 0.13	0.94

^*a*^ There was only one positive result among three assays.

^*b*^ ND, not detected.

### Detection of targets in unequal quadruple mixtures

**Fig 5:**
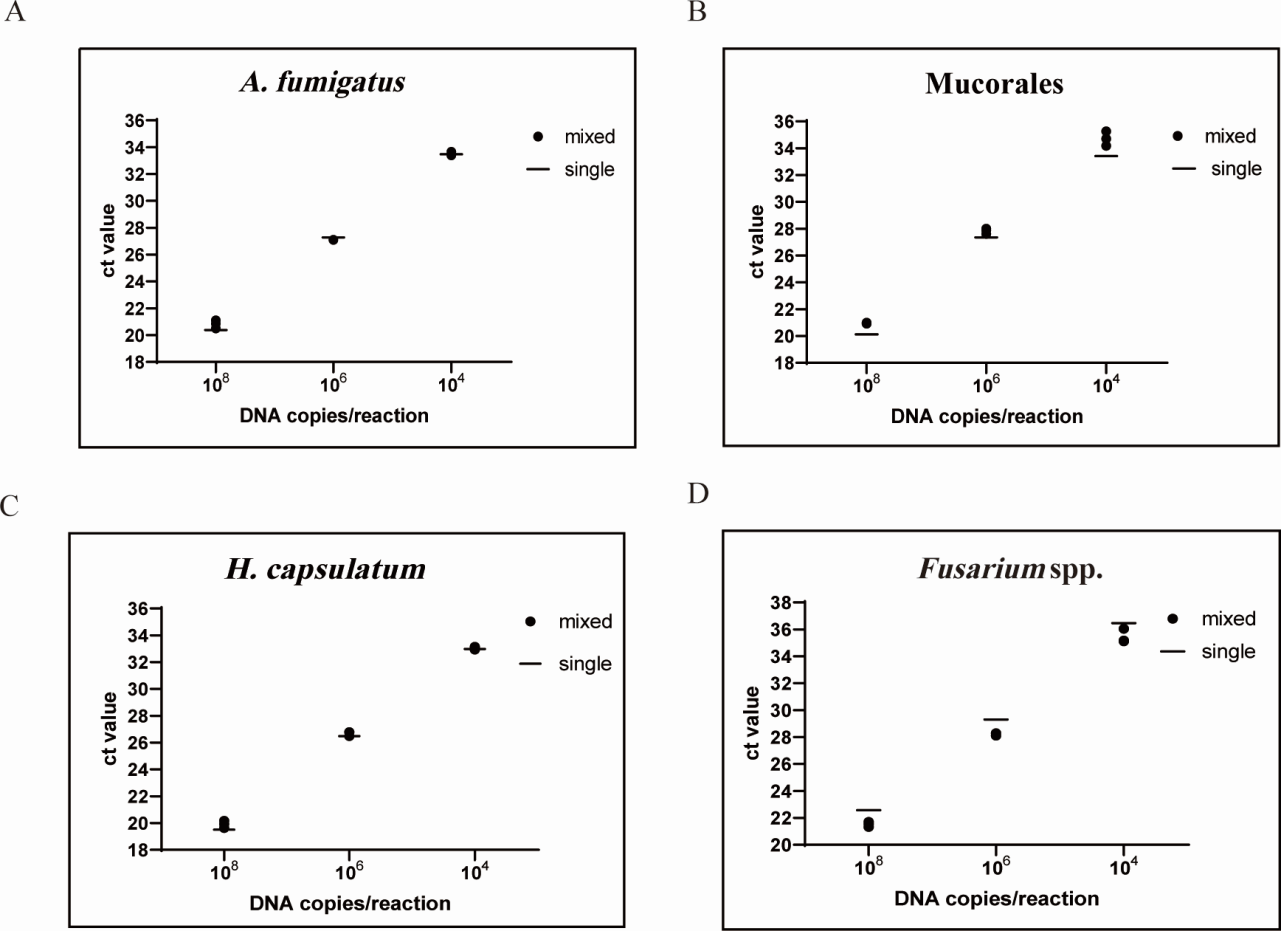
The distribution of CT values of mixed concentration detection and single concentration detection CT value. *A. fumigatus* (A), Mucorales (B), *H. capsulatum* (C), and *Fusarium* spp. (D).

To evaluate the performance of M-qPCR in detecting the mixed infections, plasmids encoding the targeted gene of four fungi were mixed to produce an unequal mixture of four targets at low, middle, and high concentrations (10^4^, 10^6^, and 10^8^ copies/µL); the specific combinations are shown in Table 4. As shown in Fig. 5, the CT value for each target in these mixed templates was similar to the CT value for that target alone, and all values were tightly clustered, with standard deviations within the range of 0.30–0.91 (*A. fumigatus*), 0.18–0.44 (Mucorales), 0.16–0.44 (*H. capsulatum*), and 0.52–0.91 (*Fusarium* spp.). Therefore, all components within a quadruple mixture, including the minority component, could be accurately quantified despite the presence of other components at different concentrations.

**TABLE 4:** Matrix for mixture compositions^*a*^

Mixture	*A. fumigatus*	Mucorales	*H. capsulatum*	*Fusarium* spp.
1	A	A	A	A
2	A	B	C	B
3	A	C	B	C
4	B	A	C	C
5	B	B	B	A
6	B	C	A	B
7	C	A	B	B
8	C	B	A	C
9	C	C	C	A

^*a*^ Orthogonal test table: A = 10^8^ gene copies/μL, B = 10^6^ gene copies/μL, C = 10^4^ gene copies/μL.

### Validation with simulated clinical samples

The simulated clinical samples comprised a 10-fold dilution series ranging from 1 × 10^6^ to 1 × 10^1^ conidia/sporangiospores of *A. fumigatus*, *M. circinelloides*, *M. racemosus*, *R. oryzae*, *R. microspores*, *F. fujikuroi*, *F. solani*, and *F. oxysporum*, ranging from 2 mg to 20 ng mycelium powder of *H. capsulatum*, mixed with 200 µL of serum or sputum. In terms of the simulated serum samples, the LoDs of M-qPCR were determined to be 10 CFU conidia/reaction for *A. fumigatus*, *M. racemosus*, *R. microspores*, *F. fujikuroi*, *F. solani, and F. oxysporum*; 10^2^ CFU conidia/reaction for *M. circinelloides* and *R. oryzae*; and 200 ng mycelium powder/reaction for *H. capsulatum*. In terms of the Sputum samples, the LoDs of M-qPCR were determined to be 10^1^ CFU conidia/reaction for *A. fumigatus*, *R. microsporus,* and *Fusarium* spp.; 10^2^ CFU conidia/reaction for *M. circinelloides*, *M. racemosus*, and *R. oryzae*; and 200 ng mycelium powder/reaction for *H. capsulatum* (Table 5).

**TABLE 5:** LoD (CFU) of simulated clinical samples tested by M-qPCR

Species	Saline		Serum		Sputum	
	LoD	CT value	LoD	CT value	LoD	CT value
*A. fumigatus*	10^1^	35.93 ± 0.47	10^1^	37.03 ± 0.84	10^1^	37.6 ± 0.58
*M. circinelloides*	10^2^	35.36 ± 0.81	10^2^	36.07 ± 0.73	10^2^	36.32 ± 0.59
*M. racemosus*	10^1^	37.34 ± 0.5	10^1^	37.1 ± 0.19	10^2^	35.04 ± 0.03
*R. oryzae*	10^2^	35.33 ± 0.46	10^2^	36.01 ± 0.45	10^2^	35.58 ± 0.39
*R. microsporus*	10^1^	36.98 ± 0.29	10^1^	36.07 ± 0.33	10^1^	37.1 ± 0.49
*F. fujikuroi*	10^1^	34.37 ± 0.28	10^1^	34.31 ± 1.17	10^1^	34.81 ± 0.02
*F. solani*	10^1^	35.34 ± 0.86	10^1^	35.48 ± 0.46	10^1^	35.7 ± 0.76
*F. oxysporum*	10^1^	35.66 ± 0.14	10^1^	35.65 ± 0.1	10^1^	35.26 ± 0.83
*H. capsulatum* ^*a*^	20	37.72 ± 0.9	200	35.57 ± 0.42	200	33.94 ± 0.16

^*a*^ The unit of this group is ng.

## DISCUSSION

In the present study, a TaqMan probe-based multiplex real-time qPCR assay was developed to quantify simultaneously and specifically the DNA from *A. fumigatus*, *Fusarium* spp., Mucorales, and *H. capsulatum*. It has been reported that *Aspergillus* spp., particularly *A. fumigatus*, are the leading cause of invasive fungal infections caused by filamentous fungi around the world, followed by *Fusarium* spp. and *Mucormycetes* (23). Additionally, in 2022, WHO announced the fungal priority pathogens list according to the clinical significance of different fungi, which classified the 19 kinds of clinically important pathogenic fungi into three levels, critical, high, and medium priority (7). *A. fumigatus*, *Fusarium* spp., and Mucorales were the most important filamentous fungi in the critical and high groups. *H. capsulatum* is a dimorphic fungus causing invasive fungal infections of the lung and systemic bloodstream infections with high mortality through inhalation of hyphae and microconidia in soil by the host (24–26). Moreover, with the increase in reports of histoplasmosis in China, the detection of *H. capsulatum* is also becoming more crucial (27). Thus, *A. fumigatus*, *Fusarium* spp., Mucorales, and *H. capsulatum* were selected for a combined and rapid detection with practical significance.

In the assay, four pairs of primer–probe sets can be mixed into one tube system, thus allowing us to detect the target pathogens simultaneously in one reaction. Remarkably, the assay has good specificity and no cross-reactivity with the other 13 pathogens. The minimum detection limit for all target species is about 1 × 10^2^ copies/µL, with a coefficient of variation below 4%, suggesting good sensitivity and reproducibility. In simulated clinical specimens, the LoD of this M-qPCR assay was about 10 CFU conidia/reaction. The primers and probes for each target of four fungi were designed on the base of S-qPCR, respectively.

For *A. fumigatus*, a number of studies have successfully detected *A. fumigatus* by targeting the conserved sequence of the membrane-bound protein *ANXC4* gene in combination with qPCR or other detection techniques (28, 29). For example, the primer designed for *ANXC4* gene in combination with loop-mediated isothermal amplification and lateral flow biosensor (LAMP-LFB) was successfully applied to the detection of *A. fumigatus*, with a minimum detection concentration of 10 fg (28). In addition, there are studies that have designed primers for the detection of *A. fumigatus* based on *ANXC4* gene, and the diagnostic accuracy was 100% (30). The 18S ribosomal RNA gene (4–6) and 28S rDNA (5, 31) have ever been targeted to detect the Mucorales, which appeared to be less suitable as a marker for the Mucorales (21). In our previous study, *rnl* gene was selected as a target to successfully detect the Mucorales by qPCR and recombinase polymerase amplification (RPA) (21). Therefore, *rnl* gene was also chosen for the detection of Mucorales in this M-qPCR assay. Since the *EF1α* gene has been targeted successfully to detect and quantify *Fusarium spp* (32, 33), the *EF1α* gene became the preferred sequence for the identification of *Fusarium spp*.; in contrast, the internal transcriptional spacer (ITS) of fungal rDNA is not suitable for species differentiation of *Fusarium spp*., although it is frequently used for fungal diversity studies. The *Hcp100* protein is a regulatory protein of *H. capsulatum* and has been proposed by many mycologists as a novel therapeutic target for histoplasmosis treatment (34, 35). Interestingly it has also been proved to be an excellent target for molecular detection and identification in clinical samples, since a combination of nested PCR and qPCR targeted to *Hcp100* gene has been designed to successfully detect *H. capsulatum* with 100% specificity (36, 37).

In this study, the primer and probe were first designed for the above four target genes and evaluated for specificity and sensitivity by S-qPCR, respectively. Afterward, they were optimized successfully and established for M-qPCR assay. In order to optimize the CT value of M-qPCR close to that of S-qPCR and evaluate the LoD of M-qPCR, the respective concentration of four primers and probes were adjusted using orthogonal experiments in the multiplex detection system. In our optimal system, the LoD for M-qPCR and S-qPCR were the same, both of which were around 10^2^ copies/reaction. However, the CT value of each target gene in the M-qPCR is slightly higher than that of S-qPCR, this may be due to the fact that multiplex PCR is a highly complex reaction that can be interfered with by mixed amplification of many targets (38).

Apparently, the sensitivity of multiplex detection is slightly lower than that of single detection in our study. This was in line with some other studies about the multiplex detection of porcine virus, in which the sensitivity of S-qPCR was as less as 10 copies in contrast to approximately 100 copies in M-qPCR. This difference was probably attributed to the competition between primers, probes, templates, and reagents in M-qPCR system (39). Moreover, different annealing temperatures of 56°C, 58°C, and 60°C were also experimented, and no significant difference was found in the detection sensitivity. However, since the specificity of M-qPCR assay could be improved by a higher annealing temperature (40), the annealing temperature was still set to 60°C in the M-qPCR assay. At the same time, the LoD value of the control group within normal saline was generally lower than that within the simulated serum or sputum; this might be due to the complex composition of sputum and serum, as well as the effect of the DNA extraction process. This result was in line with the findings in the study of a rapid detection of Mucorales by Xu et al. (21).

It is worth mentioning that unlike mammalian cells and viruses, the fungal cells are covered by a cell wall that is rich in polysaccharides and chitin, which makes the DNA of fungi difficult, and only a small amount of DNA of fungi can be obtained by normal extraction of clinical samples (41, 42). In our newly established M-qPCR system, only 1 µL of template of fungal DNA was used, which was lower than the 2 µL and 5 µL used in other studies (43, 44), and the LoD in the simulated samples was about 10 CFU/reaction. This result indicated that the M-qPCR assay possessed a higher sensitivity with less prerequisite of the amount of DNA template and the volume of clinical samples such as blood and sputum. In addition, M-qPCR can detect multiple fungal pathogens at a single time point in less than 2 hours. It was time-efficient and cost-effective; however, this M-qPCR assay needs further validation with real clinical blood, bronchoalveolar lavage fluid (BALF), or sputum samples, and the utilization of M-qPCR assay for diagnosis of fungal infection needs more advocation in clinic.

In a word, this study successfully established a TaqMan probe-based multiplex real-time qPCR assay for four clinically important filamentous fungi, *A. fumigatus*, *Fusarium* spp., Mucorales, and *H. capsulatum* with high sensitivity and specificity, which brings a promising potential for prompt and precise diagnosis against fungal infection.

## SUPPLEMENTAL MATERIAL

Supplemental tables spectrum.00634-24-s0001.docx Tables S1-S4.

ASM does not own the copyrights to Supplemental Material that may be linked to, or accessed through, an article. The authors have granted ASM a non-exclusive, world-wide license to publish the Supplemental Material files. Please contact the corresponding author directly for reuse.
