# Supplementary material for: Development of a TaqMan probe-based multiplex real-time PCR for the simultaneous detection of four clinically important filamentous fungi
Source: Microbiol Spectr. 2024 Jul 30;12(9):e00634-24. doi: 10.1128/spectrum.00634-24 (PMC11370266; doi:10.1128/spectrum.00634-24)
Supplement: Supplemental tables — Tables S1-S4. [file spectrum.00634-24-s0001.docx]

**Table S1 CT values analysis of S-qPCR reproducibility**

| **Species** | **copies/μL** | **Intra-Assay** | | | |
| --- | --- | --- | --- | --- | --- |
|  |  | **CT(mean±SD)** | **CV%** | | |
| ***A. fumigatus*** | 1×10^1^ | ND | / | | |
|  | 1×10^2^ | 36.5 ± 0.26 | 0.71 | | |
|  | 1×10^3^ | 34.35 ± 0.14 | 0.41 | | |
|  | 1×10^4^ | 30.49 ± 0.27 | 0.89 | | |
|  | 1×10^5^ | 27.02 ± 0.26 | 0.96 | | |
|  | 1×10^6^ | 23.58 ± 0.16 | 0.68 | |  |
|  | 1×10^7^ | 20.31 ± 0.21 | 1.03 | |  |
|  | 1×10^8^ | 17.1 ± 0.07 | 0.41 | |  |
|  | 1×10^9^ | 13.53 ± 0.18 | 1.33 | |  |
|  | 1×10^10^ | 9.82 ± 0.43 | 4.38 | |  |
| **Mucorales** | 1×10^1^ | ND | / | |  |
|  | 1×10^2^ | 37.79 ± 0.26 | 0.69 | |  |
|  | 1×10^3^ | 35.65 ± 0.39 | 1.09 | |  |
|  | 1×10^4^ | 32 ± 0.05 | 0.16 | |  |
|  | 1×10^5^ | 28.56 ± 0.1 | 0.35 | |  |
|  | 1×10^6^ | 25.15 ± 0.15 | 0.6 | |  |
|  | 1×10^7^ | 21.62 ± 0.07 | 0.32 | |  |
|  | 1×10^8^ | 18.01 ± 0.22 | 1.22 | |  |
|  | 1×10^9^ | 14.59 ± 0.08 | 0.55 |  |  |
|  | 1×10^10^ | 11.06 ± 0.1 | 0.9 |  |  |
| ***H. capsulatum*** | 1×10^1^ | ND | / |  |  |
|  | 1×10^2^ | 37.18 ± 0.41 | 1.1 |  |  |
|  | 1×10^3^ | 33.4 ± 0.17 | 0.51 |  |  |
|  | 1×10^4^ | 29.88 ± 0.19 | 0.64 |  |  |
|  | 1×10^5^ | 26.63 ± 0.03 | 0.11 |  |  |
|  | 1×10^6^ | 23.28 ± 0.11 | 0.47 |  |  |
|  | 1×10^7^ | 19.81 ± 0.15 | 0.76 |  |  |
|  | 1×10^8^ | 16.34 ± 0.11 | 0.67 |  |  |
|  | 1×10^9^ | 12.79 ± 0.08 | 0.63 |  |  |
|  | 1×10^10^ | 8.83 ± 0.11 | 1.25 |  |  |
| ***Fusarium* spp.** | 1×10^1^ | ND | / |  |  |
|  | 1×10^2^ | 37.51 ± 0.49 | 1.31 |  |  |
|  | 1×10^3^ | 35.37 ± 0.46 | 1.3 |  |  |
|  | 1×10^4^ | 31.5 ± 0.18 | 0.57 |  |  |
|  | 1×10^5^ | 28.11 ± 0.11 | 0.39 |  |  |
|  | 1×10^6^ | 24.56 ± 0.17 | 0.69 |  |  |
|  | 1×10^7^ | 21.1 ± 0.15 | 0.71 |  |  |
|  | 1×10^8^ | 16.32 ± 0.1 | 0.61 |  |  |
|  | 1×10^9^ | 13.89 ± 0.13 | 0.94 |  |  |
|  | 1×10^10^ | 10.12 ± 0.11 | 1.09 |  |  |

**Table S2 Matrix for quadruple mixture composition**

| Mixture | ***A. fumigatus*** | **Mucorales** | ***H. capsulatum*** | ***Fusarium spp.*** |
| --- | --- | --- | --- | --- |
| 1 | A | A | A | A |
| 2 | A | B | C | D |
| 3 | A | C | E | B |
| 4 | A | D | B | E |
| 5 | A | E | D | C |
| 6 | B | A | E | D |
| 7 | B | B | B | B |
| 8 | B | C | D | E |
| 9 | B | D | A | C |
| 10 | B | E | C | A |
| 11 | C | A | D | B |
| 12 | C | B | A | E |
| 13 | C | C | C | C |
| 14 | C | D | E | A |
| 15 | C | E | B | D |
| 16 | D | A | C | E |
| 17 | D | B | E | C |
| 18 | D | C | B | A |
| 19 | D | D | D | D |
| 20 | D | E | A | B |
| 21 | E | A | B | C |
| 22 | E | B | D | A |
| 23 | E | C | A | D |
| 24 | E | D | C | B |
| 25 | E | E | E | E |

**Orthogonal test table: A=50nM, B=100nM, C=200nM, D=300nM, E=400nM.**

**Table S3 S-qPCR specific results**

| **Species** | ***A. fumigatus*** | **Mucorales** | ***H. capsulatum*** | ***Fusarium* spp.** |
| --- | --- | --- | --- | --- |
|  | CT (mean ± SD) | | | |
| *A. fumigatus* plasmid | 24.24 ± 0.59 | **-** | **-** | **-** |
| Mucorales plasmid | **-** | 24.7 ± 1.05 | **-** | **-** |
| *H. capsulatum* plasmid | **-** | **-** | 24.65 ± 0.45 | **-** |
| *Fusarium* spp. plasmid | **-** | **-** | **-** | 24.99 ± 0.83 |
| *A. flavus* | **-** | **-** | **-** | **-** |
| *A. terreus* | **-** | **-** | **-** | **-** |
| *A. niger* | **-** | **-** | **-** | **-** |
| *C. glabrata* | **-** | **-** | **-** | **-** |
| *C. auris* | **-** | **-** | **-** | **-** |
| *C. parapsilosis* | **-** | **-** | **-** | **-** |
| *C. glabrata* | **-** | **-** | **-** | **-** |
| *P. Oxalicum* | **-** | **-** | **-** | **-** |
| *P. chrysogenum* | **-** | **-** | **-** | **-** |
| *S. aureus* | **-** | **-** | **-** | **-** |
| *E. coli* | **-** | **-** | **-** | **-** |
| *P. Oxalicum* | **-** | **-** | **-** | **-** |
| *P. chrysogenum* | **-** | **-** | **-** | **-** |
| A549 | **-** | **-** | **-** | **-** |

**-, not detected**

**Table S4 M-qPCR specific results**

| **Species** | ***A. fumigatus*** | **Mucorales** | ***H. capsulatum*** | ***Fusarium* spp.** |
| --- | --- | --- | --- | --- |
|  | CT (mean ± SD) | | | |
| *A. fumigatus* plasmid | 24.24 ± 0.97 | **-** | **-** | **-** |
| Mucorales plasmid | **-** | 25.29 ± 0.31 | **-** | **-** |
| *H. capsulatum* plasmid | **-** | **-** | 25.95 ± 2.47 | **-** |
| *Fusarium* spp. plasmid | **-** | **-** | **-** | 26.13 ± 1.03 |
| *A. flavus* | **-** | **-** | **-** | **-** |
| *A. terreus* | **-** | **-** | **-** | **-** |
| *A. niger* | **-** | **-** | **-** | **-** |
| *C. glabrata* | **-** | **-** | **-** | **-** |
| *C. auris* | **-** | **-** | **-** | **-** |
| *C. parapsilosis* | **-** | **-** | **-** | **-** |
| *C. glabrata* | **-** | **-** | **-** | **-** |
| *P. Oxalicum* | **-** | **-** | **-** | **-** |
| *P. chrysogenum* | **-** | **-** | **-** | **-** |
| *S. aureus* | **-** | **-** | **-** | **-** |
| *E. coli* | **-** | **-** | **-** | **-** |
| *P. Oxalicum* | **-** | **-** | **-** | **-** |
| *P. chrysogenum* | **-** | **-** | **-** | **-** |
| A549 | **-** | **-** | **-** | **-** |

**-, not detected**
